# Propranolol in Use for Treatment of Complex Infant Hemangiomas: Literature Review Regarding Current Guidelines for Preassessment and Standards of Care before Initiation of Therapy

**DOI:** 10.1155/2013/850193

**Published:** 2013-05-20

**Authors:** Andreas Fette

**Affiliations:** Medical School, University of Pécs, Hungary

## Abstract

In 2008, the positive effects of propranolol on infantile hemangiomas (IH) have been discovered serendipitously by Léauté-Labrèze and her coworkers. Since then, propranolol has been in use in allday clinical practice worldwide for treatment of IH. It even caused some kind of paradigm shift in the overall management of these lesions, though propranolol is still not FDA approved, respectively, in “off-label” use for this indication in the majority of institutions. Thus, the aim of this communication is to evaluate the literature for current evidence regarding guidelines for preassessment and standards of care before initiation of therapy.

## 1. Introduction

Hemangiomas are one of the most common benign vascular tumors appearing in 1.5–3% of all newborns and in up to 10–12% of all (white) infants within their first year of life [1–6].

Infantile hemangiomas (IH) in general are not present or minimal at birth. During early infancy, they undergo a characteristic rapid growth, followed by a slower growth and by gradual involution [1, 3, 4, 6].

These hemangiomas are mainly located in the head and neck area. An area that only represents 1/7 of the total infant's body surface area; but because of the overall visibility and potential disfigurement, their potential functional and psychosocial impairment is always disastrous for the child's body and soul. Particularly, if orifices like eyes, ear, nose, or mouth are involved [1, 2, 4, 6–11]. 

Proposed treatment modalities in medical textbooks and literature review are various. Briefly, they include chemotherapy (like vincristine or cyclophosphamide) [12–14], Interferon alpha 2a (an inhibitor of angioneogenesis) [9, 15–17], steroids (administered either systemic or intralesional) [15, 17], radio- [12, 18, 19] or cryo-therapy [13, 16, 20–22], therapeutic embolization [9, 16, 18, 23], laser application [5, 16], and surgery [22, 24]. 

Steroids have been considered as first-line treatment by many authors, especially before the propranolol era. But steroid therapy has significant adverse effects, including weight gain, cushingoid facies, hypertension, adrenal suppression, hyperglycemia, immunosuppression (necessitating delayed immunization), gastric irritation, behavioural changes, and transient diminished longitudinal growth [8, 9, 12, 13, 15, 16, 18, 25–29]. 

Propranolol, a well-known antihypertensive drug, has been serendipitously noted to control the growth of hemangiomas by Léauté-Labrèze and her coworkers in 2008, while being in use for treatment of (congenital) heart disease in French children [19, 30–33]. 

Currently under clinical trials, propranolol appears to be very effective in the treatment of hemangiomas, by inducing fast and substantial regression of the lesions with minimal adverse effects [6, 11, 19, 28–66]. 

To date, multiple theories have been presented to explain the mechanism of action of propranolol: Storch and Hoeger summarized, that propranolol might interfere with endothelial cells, vascular tone, angiogenesis, and apoptosis [41]. In addition, many effects of propanolol on IH are proposed to be attributable to its three different pharmacological targets: vasoconstriction, inhibition of angiogenesis, and induction of apoptosis as well [28, 31, 32, 41, 52].

Indications listed so far for propranolol therapy are large (facial) hemangiomas with endangering localization and large segmental hemangiomas on the trunk and extremities [6, 11, 28, 31, 33, 41, 44, 52]. However, treatment should start as early as in the proliferation phase [28, 58].

Listed contraindications are preterm and newborn babies within 2 weeks of life, congenital heart disease with contraindications for *β*-blocker therapy, infants with episodes of obstructive bronchitis, central nervous system (CNS) disorders, and compromised renal function [28, 59].

Before initiation of treatment, a clinical examination, baseline echocardiography and 48-hour hospitalization, and respectively frequent home nursing visits to monitor vital signs and blood glucose levels have been proposed as standard of care. This includes regular reevaluations in due course, both, on an in- or outpatient basis [6, 31, 33, 52].

In actual protocols, propranolol is administered with a starting dose of 0.5 to 3 mg/kg body weight per day, divided into 2 or 3 doses [6, 11, 28, 31, 33, 41], and gradually tapered over a period of 2 weeks [31], respectively, until the target dose has been finally reached [11]. According to the age of the child, propranolol should be administered for 4 to 8 months [28, 33], or until the end of the proliferation phase [41], respectively, until complete resolution [52].

Bradycardia, hypotension, bronchospasm, wheezing, hypoglycemia, and electrolyte disturbances are potential serious adverse effects, that could result out of propranolol use [6, 11, 28, 33, 37, 40, 41, 52]. In the majority, they have been reported as rare, mild, and transient [6, 11, 28, 33, 41]. Minor side effects like sleep disturbance (i.e., restless sleep, nightmares) and gastrointestinal discomfort are more common [52].

In short summary, propranolol has become the arising “miracle drug,” after its effects on IH have been discovered serendipitously in 2008. Besides its impressive efficacy, propranolol has shown a very limited toxicity profile, which both even caused a “paradigm shift” in the management of hemangiomas worldwide. But we still have to consider that, despite of more than a 100 Medline/PubMed citations for “propranolol and hemangioma,” this propranolol is still used in allday clinical practice without any prospective trials, standards of care, or clear definitions of response [6, 28, 64]. This means that actually propranolol is still under “off-label use” in most institutions worldwide, respectively, not FDA approved for treating hemangiomas [11, 28, 39, 42, 47, 59, 64, 66].

Then like many other drugs, even when they have already been recruited by the clinicians for the use in children, pharmacokinetic data for this specific population are lacking, meaning that no prospective, controlled studies describing optimal dosage, or how to monitor side effects are available [34].

Syndromic children (e.g., PHACES, PELVIS) have been reported to be at a higher risk for cardiopulmonary complications such as bradycardia and hypotension or bronchospasm, especially in patients with atopic predisposition or underlying spastic respiratory disease. Or, hypoglycemia in low-birth-weight newborns, children with low caloric intake or diabetes and electrolyte disturbances [29, 34, 40].

 Such a fast and early adoption of this treatment modality by “nonfamiliar” specialities certainly warrants a careful monitoring, a close liaise with the pediatric cardiologist, and a sound treatment algorithm or guideline to minimize and manage potential adverse effects [34].

Thus, this publication aims to critically appraise the current evidence of guidelines and standards of care regarding basic preassessment, monitoring, and avoidance of potential adverse effects during initiation of propranolol treatment for hemangiomas in children.

## 2. Method

The keywords “(infantile) hemangioma” and “propranolol” primarily have been considered in our literature search, when being recognized within the (abstract) title or abstract. They have been either combined to the corresponding main keyword, or used individually. Second, additional terms like “monitoring,” “guidelines,” “protocols,” “standards of care,” and “recommendations/LEITLINIEN” from working groups “vascular malformations” have been applied. Third, reference lists from all retrieved articles have been searched manually for other informative terms like “preassessment,” “safety profile,” or “adverse effects.”

The literature search included hemangiomas of all types and locations, but excluded hemangiomatosis, syndromatic (i.e., PHACES, PELVIS) and venous malformations' patients.

The search has been limited to oral propranolol intake and pediatric patients; however, other treatment modalities (i.e., steroids, laser), before, after, or next to propranolol therapy have been accepted. Mainly, “PubMed” and “Medline” publications, but also “Leitlinien der Fachgesellschaften/guidelines from working groups “vascular malformations” have been taken into consideration. 

Finally, this search strategy, by focusing on the main “key words,” have generated a basic reference list from which recent and actual guidelines and standards of care regarding initiation of propranolol treatment for IH have been created, completed by application of the above-mentioned terms, and “cross-reading” or “backward chaining” from reference lists extracted from these publications.

This search strategy seems to have detected all pertinent papers and valuable information, that need to be considered, but might be prone to publication and selection bias. 

## 3. Literature Review

Finally, 39 communications have been selected for critical appraisal. They have been classified into:

- case reports and small case series with less than 12 patients (*n* = 9);
- retrospective studies and larger case series with more than 12 patients (*n* = 11);
- communications, letters, and reviews (*n* = 8);
- Leitlinien, current concepts, and consensus conference notes (*n* = 4);
- prospective studies (*n* = 3);
- meta-analyses (*n* = 2);
- randomized control trial (*n* = 1);
- clinical trial (randomized, controlled, multidose, multicenter, adaptive phase II/III study in infants) (*n* = 1).

The results will be compared to Vlastarakos and coworkers publication, in which they presented and analyzed one multicenter retrospective cohort study, two prospective cohort and two retrospective studies, six case series, and six case reports. Two of these studies represented Level II, three studies Level III, and twelve studies Level IV evidence [64]. At the end of the literature review, strength of evidence of the selected papers will be evaluated and compared to evidence levels for guideline development taken out of Vlastarakos et al. and Shekelle et al.'s tables (see Table 1) [64, 67].

The paper from Léauté-Labrèze and coworkers from 2008 is considered as the initial publication describing propranolol in treatment of IH. In this first publication, Léauté-Labrèze and coworkers administered a dosage of 2 mg/kg body weight per day of propranolol with an impressive therapeutic, but no adverse side effects in their collective of 11 patients [32].

The authors do not report in detail on any basic assessment, cardiac workup, or monitoring before initiation of treatment, but since the vast majority of their patients have been treated in the first instance for their heart disease, it could be considered as extensive. 

In the same year, Lawley et al. published two cases suffering bradycardia and occult hypoglycemia after propranolol intake of 2 mg/kg per day, given in two or three doses. Preassessment included baseline vital signs like pulse and blood pressure, fingerstick blood glucose, ECG, and echo [34]. According to our best knowledge, this has been the first report on adverse effects of propranolol in hemangioma treatment. And that despite the gross general experience these authors had in using propranolol in infants, as already mentioned in the previous paragraph.

Two years later, Naouri et al., indeed, reported no side effects in their four patients after using a similar dosage and preassessment regimen [39]. This has been in coincidence with the results of another two reports published in the following year by Da Cruz Ferreira et al. [44] and Kim and coworkers, respectively [50].

In contrast, two other authors in previous years continued reporting serious adverse effects like hyperkalemia, or hypoglycemia and hyperthermia, despite using a comparable dosage and preassessment regimen [37, 40].

Incidents of propranolol ingestion and accidental overdosage in small children have been reported, too, fortunately causing no serious adverse effects [48, 49]. Here, Love and Sikka have stated, that, until recently, there have been no clear guidelines published in the literature, about how to direct the care of small children in case of propranolol ingestion (author's note: respectively therapy). However, a monitoring comparable to the basic cardiac preassessment mentioned already above is suggested in general, if significant beta-blocker overdose or toxicity has to be suspected [48].

Evidence of case reports are usually considered as anecdotal and low (levels IV to V) because the sample size is usually small, and the reporting is always of retrospective nature. In addition, there is no clear definition of selection criteria or how to distinct the interference of other therapies or comorbidities. Thus, in my opinion, the “hyperkalemia” or “hypoglycemia” reported in these distinct case reports might have been caused by the concomitant medication as well.

However, at that stage after introduction of a “new” treatment, the “scientific content” of case reports reflects the current “standard of care,” and might form the basis for further studies and research. But it should not be forgotten, that, since these studies have been claiming that a new “revolutionary” method for the management of infantile hemangiomas has been discovered, publication bias and preselection observer bias have to be considered.

In the upcoming larger case series or retrospective cohort studies (with sample sizes from *n* = 13 to *n* = 71) published over the next years, no significant changes in propranolol dosage or preassessment standards before initiation of therapy could be observed in comparison to the data already published in the previous papers. And, all authors still continue to accept other systemic or topical treatment modalities before, during, or after propranolol treatment [29, 33, 38, 43, 45–47, 51, 55, 58, 62]. 

In summary, six working groups reported no adverse effects after propranolol intake in their patients' collectives [29, 38, 46, 47, 55]. Two groups experienced adverse cardiopulmonary effects like hypotension in their collectives [33, 45]. Bradycardia as adverse effect have been reported only once [56]. Wheezing and bronchial hyperreactivity, respectively, has been mentioned in four papers [33, 45, 58, 62]. Hypoglycemia, constipation, and cold extremities have been added by de Graaf et al. [45], while Hsu and coworkers added a “pale looking” child [62]. Other authors reported somnolence, and/or restless sleep, or nightmares as potential adverse effects in their patients [43, 45, 58].

Regarding their “medium” sample size of only 28 patients, de Graaf et al. have experienced quite a high rate of adverse effects in one single cohort of patients. So far, this group has been the only one stating that these high rate of adverse effects are recognized to be dose independent as well [45]. Zvulunov et al., however, reported only 4 adverse reactions in their 49 patients' collective [58].

Due to the lack of randomization and their retrospective nature, cohort or case controlled studies in general are ethically easy and inexpensive to perform. However, they are prone to diagnostic or therapeutic suspicion bias. Types of observer bias related to possible misclassification of the outcome, since the author might have been already influenced by preconceived beliefs of what exposure should produce which outcome.

Level of evidence changed from IV to III according to Vlastarakos and coworkers classification [64], while strength of recommendation by category of evidence for guideline development changed from D to C according to Shekelle et al. [67] and Vlastarakos et al. [64]. 

“Communications,” “letters,” “reviews,” or “updates” published within this second time period continued to recommend a dosage of 2 mg/kg propranolol per day and a baseline cardiac workup as “standard of care” [11, 36, 42, 54, 56]. This included the contributions of Léauté-Labrèze and coresearchers, the authors publishing first on the topic “propranolol in treatment for hemangiomas in infancy” [19, 30–32].

Previously or in-parallel other administered systemic or topical therapies have still not been excluded in order to avoid potential interference or therapeutic bias [11, 36, 42, 54, 56]. 

In some of these communications, adverse effects have been reported or recitied by the authors out of other literature reviews. In the majority, these adverse effects have been minor in nature. Serious effects, like cardiovascular or hypoglycemic events, have not been detected [11, 19, 36]. In 2011, it has been for the first time, that the primary authorship, the Léauté-Labrèze research group, took adverse effects after the administration of propranolol in treatment of infantile hemangiomas into closer consideration [19]. 

In their most recent publication, an “update”, Chen et al. in 2013 provided a summary of these findings and “standards of care” [65].

Traditionally, at that stage “Leitlinien”, “current concepts,” and “consensus conference” notes are released to reflect on the (pending) “standards of care” and to support the health care community with guidelines for their daily clinical practice [28, 52, 59, 66]. At their time of release, “common sense” on propranolol dosage, basic preassessment and management of potential adverse effects existed in this type of communications in a way that they are rather different than just “copy-paste paragraphs” of each other.

In an actual contribution of Drolet et al., a report of a consensus conference, a target dose of propranolol of 2 mg/kg per day with 3x equal doses has been recommended by the majority of the participants. This 3x daily dosing, with a minimum of 6 hours in between doses, has been thought to balance considerations of safety, efficacy, and convenience for the patient best [66].

For preassessment, before the initiation of propranolol treatment, an ECG has been obtained in 81%, blood pressure measurements in 41%, and an echocardiogram and the heart rate, respectively in 38% of patients each. A cardiologist consultation has been “obtained always” by 34% and “never” by 25% of the responders, respectively. Routine screening of serum glucose levels has been “not indicated” [66].

The most frequently reported serious complications by this group have been hypotension, pulmonary symptoms related to direct blockade of adrenergic bronchodilatation, hypoglycemia, bradycardia, and hyperkalemia, for which no (essential) additional details have been documented, unfortunately [66]. Minor side effects reported have been sleep disturbances including nightmares, somnolence, cool or mottled extremities, diarrhea, and gastroesophageal reflux/upset [66]. 

The real evidence of this sort of publications is hard to assess because they are under continuous development and constant updating. In the majority, they also rely on different and various data sources. However, they seem to have a considerable impact in allday clinical practice, on what will be considered as “standard of care,” respectively in formulating guidelines.

For level II evidence, followed by strength of recommendation “B” by category of evidence for guideline development, prospective comparative studies, systematic reviews of Level II studies, or Level I studies with inconsistent results and lesser-quality randomized control trials, respectively (i.e., <80% follow up, no blinding, or improper randomization) are requested, according to Shekelle et al. and Vlastarakos et al. [64, 67]. 

Therefore, 3 prospective trials have been evaluated. Each group has been using a target dose of 2 mg/kg propranolol per day [57, 60, 61], divided into 3 equal daily doses in the trials of Zaher et al. and Bettloch-Mas et al. [57, 60], respectively, and 2 equal daily doses in the trial of Georgountzou et al. [61]. 

Cardiac workup before treatment initiation has been considered as standard of care in all 3 studies [57, 60, 61].

Blood glucose levels have been determined only in 2/3 of these prospective trials [60, 61]. 

Only in Bettloch-Mas et al.'s study, propranolol has been administered exclusively in order to avoid biased results by other medications or treatment modalities. However, finally Bettloch-Mas' research group, but also the research group of Zaher, did not face any adverse effects in their collectives [57, 60]. 

In a single institution a randomized, double-blind, placebo controlled, parallel-group trial has been conducted between June 2009 and December 2010. A dosage of 2 mg/kg propranolol per day divided into 3 doses has been reported as standard [68].

Preassessment before initation of propranolol therapy has comprised cardiac workup, ECG and echocardiogram, full blood count, electrolytes and liver function tests, and blood glucose levels. These laboratory investigations have been performed as baseline investigations in a sense of precaution, since this has been a clinical trial as well [68].

Although some of the patients received other treatment modalities, no episodes of hypotension, bradycardia, or hypoglycemia have been observed during careful monitoring of blood pressure, heart rate, and blood glucose levels. Briefly, the adverse events included bronchiolitis, upper respiratory tract infection, and sleep disturbance with crying episodes possibly due to nightmares, and one child experiencing transient cool extremities. Their sample size comprised 40 children with hemangiomas at risk to cause disfigurement, or hemangiomas that have failed to respond to corticosteroide therapy. Patients have been randomly assigned to receive either propranolol or placebo [68].

In a randomized clinical trial, a predefined human population with a defined problem is randomly allocated to two or more comparable treatment conditions for a defined period of time. Outcomes are then compared between the groups. RCTs in general are the accepted “gold standard” of medical research. When conducting RCTs, researchers have to observe stringent methodological guidelines based on sound scientific reasoning. This finally safeguards the robustness and validity of the results found [69].

 Hogeling and her coworkers concluded that the result of their RCT have revealed that “propranolol is a safe and effective medication for treating IHs” (with statistically significant *P* values) [68].

Limitations stated by the authors have been the heterogenous patient population and their small numbers of patients (*n* = 40) [68]. (Author's note: not to forget the different anatomic sites of hemangioma localization and their stages).

Hogeling et al. further concluded that it is still evident to perform larger multicenter trials to gather more detailed information (and evidence) about the safety (profile) of propranolol in this patient population [68]. 

Two meta-analyses have been conducted to date focusing on the treatment of airway hemangiomas. In both studies, propranolol finally has been tapered to 2 mg/kg per day in 3 divided doses [53, 64]. 

In the second one, Vlastarakos and his coworkers stated, that from the pharmacological point of view the optimal dosing interval for propranolol is six hour, but that better compliance will be achieved, when the medication will be given in 2 or 3 daily doses [64]. 

Both meta-analyses agreed on that baseline vital signs and blood glucose levels have to be obtained, together with a full cardiovascular and respiratory review prior to initiation of propranolol therapy. These reviews have deemed to be necessary, especially if either of the findings shows abnormal results [53, 64]. 

Peridis and her coworkers evaluated the Great Ormond Street Hospital guidelines. They recommended to perform a full cardiovascular and respiratory review, blood tests (like FBC, urea and electrolytes, creatinine, LFT, and glucose and thyroid function tests), urine dipstick for glucose, ECG, echo, and an abdominal ultrasound scan prior to initiation of therapy [53]. 

In their present meta-analysis, Vlastarakos et al. have identified four adverse effects in the children receiving propranolol: two asthmatic attacks, which had necessitated cessation of treatment, one episode of pallor without loss of consciousness, which has been attributed to a possible vagal reaction, but did not require discontinuation of treatment or dose adjustment, and a soft palate ulcer, which responded well to steroid treatment [64]. 

Peridis et al., indeed, stated bradycardia, hypotension, hypoglycemia, rash, gastrointestinal discomfort/reflux, fatigue, and bronchospasm as potential side effects of propranolol, but all of them tend to be quite rare and have been seen at doses > 2 mg/kg/per day [53]. (Author's note: please compare this to the reports on propranolol overdose in the previous paragraphs).

A meta-analysis is a statistical summarization of study results, that has been conducted on a specific clinical question. This means that a meta-analysis is pooling the results from several studies to produce an overall estimate of the effect size. Results are usually presented with *P* values, confidence intervals and are displayed on a forest plot. Although details of the meta-analysis calculations are beyond the scope of critical appraisal, it is worth noting that meta-analyses are used to explore and analyse the various effects of random and systematic error, that are seen to have occurred in the individual studies. A meta-analysis procedure ensures that these errors are identified and to an extent adjusted and corrected prior to computing an overall estimate. In a meta-analysis of results from several studies, any variability seen to occur from study result can be ascribed either to chance, systematic differences, or both [70].

The main aim of the present meta-analysis of Vlastarakos et al. has been to critically review the current evidence on the efficacy of propranolol in the management of airway hemangiomas [64].

Adverse events and treatment failures have also been explored included in a secondary end-point analyses of a protocol for the safe administration of propranolol and appropriate patient follow up [64]. 

Finally, the authors concluded that high-quality evidence is still not available. Their results suggested that propranolol can be recommended for the treatment of airway hemangiomas, since it has been found to be effective and outperformed the previously considered “gold standard” methods of treatment (*P* < 0.001) with fewer side effects [64]. Regarding a protocol for the safe administration of propranolol and an appropriate patient follow up after the initiation of therapy, no more evidence-based data have been provided. Just the comment that “active parental monitoring is also essential to ensure treatment safety” [64]. 

A randomized controlled multidose multicentre adaptive phase II/III study in infants has been started in the United States in 2012. This study has been designed to confirm the efficacy of propranolol in severe IH by demonstrating superiority over placebo, as well as to document the safety profile of administering propranolol for this indication [63]. 

Inclusion and exclusion criteria together with selection of type of hemangiomas and avoidance of interference with other treatments are much more strict compared to the other study designs [63]. Thus, we might be able to expect more statistically robust data in the near future. However, because of the strict exclusion criteria in this study design, many of the meaningful clinical data taken out of the former case reports or other studies will not be comparable anymore.

## 4. Discussion 

In general, all included papers on treatment of infantile hemangiomas with propranolol reported favourable results with low adverse effects, making the reports prone to publication and (diagnostic or therapeutic) suspicion bias.

Only a certain number of authors stated that the use of propranolol for this indication is still “off-label” and not FDA approved [28, 39, 42, 47, 59, 64, 66].

None of the included reports did have stringent inclusion or exclusion criteria regarding type or location of the hemangioma treated. Other treatment modalities, before or during initiation of propranolol treatment, have been generally accepted so far, though this could lead to interference bias and probably misleading results, even in trials where randomization is of key importance. Here, the actual started NCTO 01056341 US clinical trial will be based on more stringent criteria and therefore more likely to provide more robust and evidence based statistical data in the near future [63]. 

Regarding our aim to find or develop a uniform and accepted “guideline” or “protocol for treatment” in daily clinical practice, Da Cruz Ferreira et al. stated in 2011 that “we did not follow any specific protocol for treatment, because at the time we treated this first baby there were no protocols” [44].

Bagazgoitia et al. stated in the same year that “the variability in all these measurements was due to hospitalization policies in the different hospitals in the study” [43].

And Missoi et al. added that “debate has taking place regarding the most favourable method for safe initiation and monitoring of treatment with oral propranolol” [51].

Bertrand et al. stated that “the overall safety profile of propranolol appears positive, but long-term data are lacking” [29].

The excerpt out of all these statements might be that despite the variety of protocols and guidelines we have to face, the clinical results are very good, but real evidence is still lacking. 

## 5. Conclusion

Vlastarakos et al. admitted that “the present meta-analysis did not identify any Level I studies regarding the administration of propranolol for the treatment of proliferating airway hemangiomas” [64]. The vast majority of studies have been small case series, and the number of treated children have also been (relatively) small. However, the reported results seem to be encouraging. In addition, the uniform nature of these results, along with the quality of the studies performed, will allow to adopt a grade C strength of recommendation regarding the effectiveness of propranolol in airway hemangiomas [64]. 

Regarding the development of a guideline for preassessment and standard of care before initiation of propranolol therapy for IH, the overall nature of the results is uniform, too, but along with the quality of the studies performed, I would adopt only a grade D strength of recommendation. 

## Figures and Tables

**Table 1:** Levels of evidence (reproduced and referenced according to [64, 67]).

Category of evidence	Study design
Level I	(i) High-quality randomized trial with statistically significant difference, or no statistically significant difference but narrow confidence intervals (ii) Systematic review of Level I randomized control trials (and study results were homogenous)
Level II	(i) Lesser-quality randomized control trial (i.e., <80% followup, no blinding, or improper randomization) (ii) Prospective comparative study (iii) Systematic review of Level II studies or Level I studies with inconsistent results
Level III	(i) Case control study (ii) Retrospective comparative study (iii) Systematic review of Level III studies
Level IV	Case series
Level V	Expert opinion

Strength of recommendation	Category of evidence

A	Directly based on category I evidence
B	Directly based on category II evidence or extrapolated recommendation from category I evidence
C	Directly based on category III evidence or extrapolated recommendation from category I or II evidence
D	Directly based on category IV evidence or extrapolated recommendation from categories I, II, or III evidence

## Abbreviations

ECG: Electrocardiogram
Echo: Echocardiogram
FBC: Full blood count
IH: Infantile hemangioma
LFT: Liver function test
PELVIS: Perineal hemangiomas, external genital malformations, lipomyelomeningocele, vesicorenal anomalies, imperforate anus, and skin tag
PHACES: This association is characterized by posterior fossa abnormalities, typically plaque-like and segmental hemangiomas in the cervicofacial area, arterial anomalies, cardiac and aortic arch defects, eye anomalies, and sternal clefts or supraumbilical raphae
RCT: Randomized clinical trial.
